# Analecta Minora

**Published:** 1827-07

**Authors:** 


### ANALECTA MINORA.

### 1. SUPPOSED ANEURISM.

As this subject has lately excited considerable interest, and, we fear, led to unnecessary operations, in this country, we are induced to extract the following case, from a sensible paper on the surgical anatomy of the neck, by Dr. Horatio Jameson, published in the 36th number of the American Medical Recorder. We may premise, however, the following passage from Burns.

" A tumour appeared from behind the sternal extremity of the left clavicle : it was bigger than a hen's egg—pulsated very strongly—and produced an inequality in the pulse at the wrist, great difficulty of swallowing, and slight dyspnœa." P. 79.

The patient was treated by a surgeon as for aneurism ; but, happily, there was not then such a rage for tying carotids as now prevails. The patient got tired of Valsalva's regimen, dismissing his surgeon and his restrictions at the same time. He resumed his former exercise and diet, and the tumour gradually disappeared!

We shall now give Dr. Jameson's case.

" About two years ago I was called, by a physician of the first respectability, to examine a case of aneurism at the root of the neck, which might be disease of the aorta, innominata, subclavian, or carotid, since it was large, and occupied a considerable portion of the neck, from the trachea to the *incurvature,* and from the clavicle up as high as the thyroid cartilage, forming a very prominent and large tumour. After laying my fingers upon different parts of the swelling, through all which a feeble pulsation could be felt, I remarked to my friend that the disease was obscure. He was evidently suprized, and directed me to place the point of my finger upon the highest point of the tumour, where the skin was thin, and somewhat unhealthy in appearance. I now perceived a pretty forcible pulsation. I attempted to press the blood out of the sac, to see the effect; I easily caused the blood to retire from the projection in which the strongest pulsation existed; I felt a crackling sensation, as though the parts within were tearing; I, therefore, desisted, *but felt fully satisfied that we had an aneurism.* The advanced stage of the disease, however, and a constitution utterly ruined by intemperance, forbade all thought of operating. Measures were adopted for temporising, and a moderate pressure applied to the tumour. The patient soon died from debility."

What a pity that some of our bold *operatives* were not on the other side of the Atlantic at the above period. The patient would have been saved by ligature—or, if he died of another disease, his carotid would have been bottled, and placed in some shop-window, for the edification of surgeons. When Dr. Jameson came to examine the body, he found that this said aneurism was no other than " a diseased gland, which was situated in the layer of vessels and fat under the sterno-cleido mastoid muscle." "In its upper part, it presented, in great part, the characters of venous aneurism; and owing to this circumstance was it, that the pulsation of the carotid, which lay under it, could be felt." We recommend this case, as well as some others lately recorded in this country, to be borne in mind by the young surgeon who is determined to cut his way to the temple of fame, sword in hand.

P.S. We may here notice a new kind of ligature, which Dr. Jameson has been in the habit of using, with invariable success, for many years past.

"My ligatures have been made of common fawn-skin, often with its buff-ball on it. I prefer soft pliable pieces; cut them into shreds, about four times as wide as they are thick, or, if the leather is very soft, something wider; then, holding one end fast with the left fingers, I draw it several times pretty forcibly through the right thumb and fore-finger; by this, they acquire solidity, without becoming hard, and will be extended so as not to be more than twice as wide as thick, if small, the larger perhaps three times, so that they are, in a slight degree flat. These ligatures I apply, with the utmost facility, to the ends of arteries in amputations. They never slip. I would, however, advise, that the leather for this purpose should be dressed by immersing the skin in the brain of an animal with water, and working a portion of oil into it as it becomes dry. We can thus obtain a pliable, soft, unirritating ligature."

---

2. ANCHYLOSIS CURED BY AN OPERATION.

[Dr. Barton. Pennsylvania Hospital ]

THE following case, published in the North American Medical and Surgical Journal for April, 1827, deserves the attention of our surgical brethren. It is well known that anchylosis cannot take place till the natural structure of a joint is changed, and then we can only apprise our patients of their *irreparable* loss. If the operation, successfully performed by Dr. Barton, be applicable to other cases of a similar kind, surgery will have atchieved one more victory over disease.

*Case.* John Coyle, aged 21 years, a sailor, states that, on the 17th March, 1825, he fell into a ship's hold, and pitched upon a barrel, the force of the fall being sustained by the right hip. Violent pain and much tumefaction ensued, and he was confined to his hammock, with the limb contracted, for 18 days, when he was taken to the hospital in PORTO CAVALLO. There he remained for five months, "placed on his side, with the injured limb uppermost, drawing the thigh to a right angle with the axis of the pelvis, the knee resting on the sound side." No measures of a proper kind appear to have been taken at the hospital, and the natural result was, "a rigid and deformed limb."

Various opinions were formed, after this, respecting the nature of the injury, some conceiving it to be a dislocation, some a fracture, and others a compound of both. In October, 1825, he returned to Philadelphia, where Dr. Barton saw him. He now went on crutches, the thigh drawn up nearly to a right angle with the axis of the pelvis, and the knee turned inwards and projecting over the sound thigh, so that the outside of the foot presented forward. There was considerable enlargement round the hip, so that no accurate judgment could be formed of the exact nature of the original injury. It was evident, however, that all articular movement was irrecoverably lost, since various measures were taken in the Pennsylvania hospital, without the slighest good effect.

After a years' residence in the hospital, Dr. Barton proposed to his colleagues, Drs. Hewson and Parrish, the following operation, viz:—"to make an incision through the integuments, of six or seven inches in length, one half extending above, and the other below the great trochanter; this to be met by a transverse section, of four or five inches in extent; the two forming a crucial incision, the four angles of which were to meet opposite to the most prominent point of the great trochanter; then to detach the fascia, and, by turning the blade of the scalpel sideways, to separate anteriorly all muscular structure from the bone, without unnecessarily dividing their fibres. Having done this, in like manner, behind and between the two trochanters, to divide the bone transversely through the great trochanter, and part of the neck of the bone, by means of a strong and narrow saw, made for the purpose; this being accomplished, to extend the limb, and dress the wound —and, after the irritation from the operation shall have passed away, to prevent, if possible, by gentle and daily movement of the limb, the formation of a bony union, and to establish an attachment by ligament only, as in cases of ununited fracture, or artificial joints." 282.

The foregoing operation was actually performed before a large medical class, and with the assistance of Drs. Hewson and Parrish, on 22nd day of November, 1826. The bone being divided, the thigh was released, and Dr. B. immediately turned out the knee, extended the leg, and placed the limbs side by side. The unsound member betrayed a shortening of about half an inch. Union by the first intention was not attempted—the patient was put to bed—and Desault's splints were applied. The operation, though severe, did not last above seven or eight minutes. Considerable pain and irritation followed, and the next day the patient had inordinate vomiting, the pulse being feeble and rapid, with great pain along the forepart of the thigh. Opium and soda water—saline draughts—sinapism over the epigastrium, gave some relief. We need not follow the diurnal details. By the 1st of December, the whole surface of the wound was covered with healthy granulations. *7th Dec.* There was copious discharge of matter from the wound, and bark was given, with opium and stimulants. *21st.* Wound cicatrising—pus diminishing in quantity. *January 20th*, 1827. Strength has regularly increased—sore progressively diminished. After the 20th day from the operation, the limb was cautiously moved, in such directions as resembled the natural movement of the sound hip-joint. This was repeated and continued according to the patient's ability to bear it; but never carried to the extent of giving much pain or of leaving permanent irritation. In the course of a short time, the part became less sensible to pain from this disturbance, and the limb was more frequently moved. The patient was then directed to exert his own muscles in slightly bending, extending, and rotating the thigh. This he accomplished without difficulty, and, after a little practice, without pain. At the end of sixty days, the wound being perfectly closed, and all appearance of inflammation gone, the patient left his bed, and, aided by crutches, stood erect, both feet reaching the floor. He even bore some weight on the lame limb, and advanced the leg a little by muscular exertion alone. *Feb. 8th.* His strength is gradually increasing. To-day, he walked one hundred yards, and, with aid, got into a gig, and rode through the city. *March 1st.* Patient rapidly gains strength—appetite good—keeps up all day—amuses himself by exercise in walking, which he now does with only the aid of a cane. "The following is the degree to which he can perform the movements of his limb with perfect ease: —by measurement from a straight line, he can advance the foot twenty-four inches; in stepping backward, twenty-six inches, and outwards, six inches."

Dr. Barton has entered into an enquiry how far the principle of this operation is applicable to the formation of artificial joints in other parts of the body, where natural motion has been lost. His reflections have not presented any forbidding circumstances; but we have not space to enter fully into his arguments, and therefore shall leave the results of this ingenious, bold, and fortunate operation to the contemplation of our surgical readers.

---

3. HEPATITIS—SUPPURATION—PARACENTESIS THORACIS.*

A very interesting case of this kind is related by Mr. Huggins, of Derby, in the Medical Repository of this month, (July) of which we shall here present an extract.

*October 27th*, 1825. Mr. H. was called to a young man, 27 years of age, who complained of great pain in the right hypochondrium, extending to the shoulder and clavicle, attended with difficult respiration, and hard full pulse. He was bled and freely purged. *28th*, There was little or no relief of the pain; and bilious vomiting was superadded. There were also dry cough, and some tenderness of the abdomen. Again bled to 20 ounces,

* A case of inflammation of the liver terminating in suppuration, and discharging itself into the cavity of the chest, successfully treated by the operation of paracentesis thoracis. By Mr. G. Huggins, surgeon, Derby, Med. Repos. July, 1827.

and also purged. Twenty leeches to the right side. *29th.* The symptoms were mitigated, but the cough remaining troublesome, antimonials and a blister were prescribed. *30th.* Pulse 120—respiration extremely difficult — expectoration free. Bled again to 16 ounces, with an active purgative. *31st.* Pain greatly increased—inability to turn to the left side —frequent burning flushes. Venesectio ad deliquium—no alleviation of the pain—blood cupped and buffed. In the evening he had tendency to delirium—the eye was turning yellow—the pulse being still sharp and quick, the lancet was again put in requisition, and with evident relief. Small doses of mercury, with antimony and opium every four hours. *November 1st.* Pain diminished, but the cough remains troublesome—whole body tinged yellow. In the evening, the pain of side returned, and the patient was again bled, with great relief. The prostration of strength was now considerable. On the 3rd of November, there was some soreness of the mouth. The calomel and opium discontinued. Has some shiverings succeeded by heat. *6th.* Considerable prostration of strength. The decoct. cinchonæ was given, and the patient continued to amend till the 12th, when there was some return of pain and fever, apparently produced by agitation of mind. In a few days, however, the symptoms subsided, and he continued to improve, with the exception of his cough, which, after the lapse of a month, seemed to increase rather than decrease. There was a copious expectoration of viscid fluid. Mr. H. gave up his attendance on the patient; but in the month of February, 1826, he was summoned to the patient who was suffering from great dyspnœa, sense of suffocation, inability to lie down, copious expectoration of purulent matter, countenance pale and sallow, tongue furred, entire loss of appetite, pulse 110 to 130, gastric irritability. The chest was now examined. The right side of the thorax was found to bulge out considerably—the mamma much swollen—dull sound over all that side—no respiration to be heard through the stethoscope —on the other side, the respiratory murmur was every where audible. There was also a sense of fluctuation in the right side, the whole of which side was passive in the act of respiration. Dr. Hart was consulted, and the operation of paracentesis thoracis was proposed. On the following day (Feb. 13th) an incision was made with a scalpel between the sixth and seventh ribs. A stream of sero-purulent fluid gushed out with great force, to the amount of six or seven pints, together with some offensive gas. The breathing was much relieved, but exhaustion was considerable. He slept during the succeeding night. A small opening was left for the discharge of fluid. *Feb. 19th.* The patient was found much improved. There was a free expectoration of matter by the mouth, and a copious discharge from the wound. The decoct. cinchonæ was administered, and he progressively improved, the discharge from the wound, at the rate of a tea-cupful per diem, continuing for thirty-six weeks. Soon after this the wound healed, and the man perfectly recovered, being now at work in his usual occupations.

Percussion yields a dull sound in the right side of the chest, as high up as the fifth rib; but a clear sound above that place. The respiratory murmur is partially audible till the stethoscope reaches the fifth rib, below which it cannot be heard at all. The voice is resounding at the superior part of the chest, but there is no pectoriloquism. In the left side every thing is natural. The right side of the thorax does not expand during inspiration, in the same manner as the left.

*Remarks.* The foregoing case does great credit to Mr. Huggins. But we doubt whether there is any decisive evidence of suppuration in the liver. That there was great inflammation in that organ cannot be doubted; and that the inflammation spread to the lung of that side, there can be as little doubt. In our own opinion the seat of the suppuration was in the chest; but the operation and final termination of the case reflect the greatest credit on Mr. Huggins.

While analyzing this paper (June 9th) we met with a fatal case of hepatitis presenting some *post mortem* features different from any thing of the kind which we have ever before seen, or read of. The patient was a female, residing in Whitcomb-street, and under the care of Mr. Fincham, of Spring Gardens. She had been confined about two months previously, and had never been well since. We could not, however, get any clear history of the case, before the present illness, which commenced on the 29th May, with pain in the epigastrium, great tenderness of the abdomen, gastric irritability, and fever. Venesection and leeches were several times repeated, with blisters, purgatives, and all the usual means; but with no relief. She turned as yellow as an orange on the third day of her illness, and the liver could be felt far down in the abdomen. Three or four days before her death (8th June) she had violent rigors, denoting suppuration, and the fever became greatly moderated.

On dissection, there was universal peritoneal inflammation and adhesions. The liver descended below the umbilicus, and was intimately adherent to the peritoneum lining the anterior parietes of the abdomen, and to the stomach, colon, and small intestines, on its concave surface. There was such a complete chaos of adhesive inflammation and agglutinated parts about the gall-bladder, that we could not disentangle it; and it was burst in the attempt. A large quantity of thin green bile flowed out, and it was a long time before we could soak it all up with sponges. When the liver was cut into, the same kind of fluid gushed out from every part, so that there must have been at least a pint and a half of green bile in the substance of the liver itself. The fluid was contained partly in the ducts, some of which were so dilated as to be capable of receiving the point of the finger—partly in rents or depôts in the parenchymatous structure of the organ, into which the fluid had been extravasated from the torn ducts. The whole of the liver was thus like one vast honeycomb—the cells communicating with the biliary tubes, and these last dilated to a most extraordinary and incredible size —all filled with the same kind of green bilious fluid which was found in the gall-bladder. With some difficulty we took away a portion of liver, and have shewn it to many medical gentlemen, none of whom ever saw a similar specimen of morbid anatomy. We regret that the state of the parts and the hurried manner in which we were obliged to make the dissection, (in the presence of the sister of the deceased,) prevented our ascertaining whether the ductus communis choledochus was, or was not permeable. We think, from the symptoms, that it had for many days been impervious, by violent inflammation.

---

### 4. HERNIA, WITH RETENTION OF URINE AND RUPTURE OF THE BLADDER.

A case of this kind occurred lately at St. George's Hospital, in the practice of Mr. Rose. The following is a concise account of the particulars.

John Culver, æt. 52, was admitted May 30th, 1827, having pain and tenderness over the pubes, with frequent and painful micturition. He was a very old patient in the hospital, having entered it originally for stricture. About four years ago he was seized with retention of urine, for which the bladder was punctured by Mr. Gunning. Eighteen months ago, he had a second attack of retention, and Mr. Rose was obliged to puncture again. For a considerable time past it had been found impossible to get an instrument into the bladder, in consequence of its entering a false passage, which appeared to lead to some depth behind the prostate. This being the case, and the patient suffering excruciating pain from each attempt at introduction, the urethra was left undisturbed. The urine was voided frequently, in small quantities, and at times with very severe pain. The man was employed about the hospital, but not retained as a patient. *Hirud.* x. *regioni pub. haust. salin. c. tinct. opii*, gtt. xxv. *vesp.*

On the next day the symptoms were much relieved. June 3d. Whilst straining to make water, a rupture took place along the inguinal canal, on the right side. The house-surgeon reduced the gut, though with some difficulty. June 3d, 1 p. m. In the hypogastric and right iliac region, there is an ill-defined tumour, exquisitely painful on the slightest touch. He has not made water since 3 o'clock, A. M. and it seems that he has had no motion since the 1st. Great anxiety—pulse quick and bounding. The warm hip-bath was tried, but gave no relief. As it seemed probable from the retention of urine, and exquisite tenderness of the hypogastrium, that urine had got extravasated into the cellular membrane, it was determined to cut down upon the tumour and ascertain its nature. An incision was accordingly made into it, in the line of the fibres of the external oblique, and a second from the middle of this in a direction towards the umbilicus. The cellular membrane over the lower part of the recti muscles was found to be in a sloughy state, and some fluid having a decidedly urinous smell escaped. *Fomentations.* 9 p.m. The bowels have acted twice, and the water passed freely both from the wound and urethra. *P. ipec. comp.* gr. xv. *cal.* gr. v. *statim—haustus sennæ cras mane.* 5th. Extremely low to day—skin cold and clammy—pulse quick but small—he is sick at times. There is perceptible emphysema about the wound. *Vesp.* The sickness and depression still continuing, and the bowels not having been freely opened, Mr. Rose thought it possible that the hernia had not been entirely reduced. To decide this point, Mr. R. determined to operate, on doing which he found a small knuckle of intestine down, and quite black. The stricture, which was at the inner ring, was divided, and the gut returned into the abdomen. The patient, however, did not rally, and in spite of the exhibition of brandy, carbonate of ammonia, and opium, he sank at 9, p.m. next day.

*Dissection.* On opening the abdomen the viscera were found glued together by a mass of old and recent adhesions. In a portion of ileum, was seen the small knuckle of intestine which had been strangulated; it was a good deal congested, but otherwise sound. There was pus in the cavity of the pelvis. Attention was next paid to the state of parts about the bladder and pubes. A probe could be passed from the sloughy abscess in the hypogastrium down to the anterior part of the bladder, below its fundus, and on the outside of the peritoneum. The organ itself was much thickened and diseased, and on laying it open, it was evident that the cicatrix of the original wound, made by Mr. Gunning, had given way. The first two or three inches of the urethra were quite sound, but near the membranous part the false passage commenced, and proceeded to some distance behind the prostate, where it gradually lost itself in the cellular membrane. It was lined by an adventitious mucous membrane. Just anterior to the caput gallinaginis was a stone the size of a large almond, and lodged in an abscess, partly bounded by the body of the prostate, but principally situated in the membranous portion of the urethra. Beyond this the canal appeared to be entirely obliterated, but, at length, a small opening was with difficulty discovered, which, by means of a fine probe, was found to lead into the false passage, thus keeping up the communication between the bladder and urethra.

*Remarks.* It is curious to follow out the progress of this complicated case. Affected originally with stricture, retention of urine came on, and to the attempts at introducing an instrument for its relief, we may, in all probability, ascribe the commencement of the false passage. The water was now voided with extreme difficulty, and its accumulation in the bladder, together with the chronic inflammation set up in that viscus, was, no doubt, the main cause of the deposition of the stone. Where this was formed it is difficult to say; it is evident that, lodging in the membranous part of the urethra, it became an additional obstruction to the passage of the urine. The stone increased in size, and the cavity in which it lay was proportionately enlarged by ulceration. The false passage too was dilated by the introduction of instruments, whilst the natural channel became almost obliterated. In this state of things, it is not to be wondered at that retention of urine should take place, or that, having taken place, one of the old cicatrices in the bladder should give way, and the urine get extravasated into the cellular membrane. This, of itself, would most probably have killed the patient, broken down in constitution as he was, but when hernia also supervened, the case was hopeless indeed.

---

### 5. FRACTURE OF THE PELVIS, WITH RUPTURE OF THE URETHRA.

[St. George's.]

Nicholas Glynn, æt. 32, was admitted into St. George's Hospital, June 9th, at 4, p.m. under the care of Mr. Jeffreys, with simple fracture of both thighs. The accident happened about an hour previously. Some men were employed above him in carrying a considerable weight; it slipped and precipitated him down a flight of twelve stairs. There were several wounds and bruises about the arm and shin—pulse small and weak—surface cold and exsanguined. The thighs were put up in Dessault's splint. In the evening there came on pain in hypogastrio, and the patient complained of not being able to pass his urine; no tumour, however, could be felt in the region of the bladder. A large-sized gum catheter was introduced, to all appearance into the bladder, and six ounces of fluid blood drawn off. *A draught of sp. æth. sulph.* ʒj. *tinct. opii,* ♏ xxx. *and aq. menth. pip.* ℥iss. *every three hours.* At night, the pain in the hypogastrium increased, the countenance became anxious, and there was a cold perspiration on the skin. He vomited the draught. Two or three ounces more of bloody fluid were drawn off by the catheter. A little brandy and water occasionally.

10*th.* Very ill indeed—the pulse is small, quick, and hard—the countenance extremely anxious—thirst—constant vomiting. The pain in the belly is horrible, and has extended more towards the navel. Some fulness perceptible in the right iliac region. Four ounces of fluid, less bloody than before, were drawn off. *Haust salin. efferv.* ℥ij.—*tinct. opii,* ♏ x. 2*dis. horis.* —*hirud.* x. *hypogastrio*—*fotus communis.* 6 *p.m.* The pain was relieved for a short time by the leeches, but it has since returned; catheterism—6 ounces of bloody fluid abstracted. *Enema, calomel, and colocynth, at night.* 11th. He is evidently sinking to-day—pulse scarcely to be felt —tumefaction at the lower part of the abdomen more general, whilst the pain has greatly subsided. The patient is sensible, though rather hurried in his manner. Tongue of a dead, dirty white, and browner in the centre. Constant vomiting of a nauseous, feculent looking fluid. At 4 p. m. he died.

*Dissection.* No marks of contusion observable about the pelvis externally. On cutting through the recti muscles, there escaped a quantity of bloody fluid, having an urinous smell, and exactly resembling in appearance that brought away by the catheter. It was contained in the cellular membrane, between the muscles and peritoneum, and for some distance around there was considerable extravasation of blood. The cellular tissue itself, in which the fluid was collected, was in a sloughy state. On exposing the cavity of the pelvis, it was discovered that the pectineal portion of each os pubis, where it forms the upper boundary of the thyroid foramen, was broken; on the right side the bone was shattered. The bladder was opened; but was quite healthy, and contained perfectly clear and limpid urine. On slitting up the urethra, a laceration was discovered in its membranous portion, immediately anterior to the prostate gland. This laceration evidently communicated with the cavity above-mentioned beneath the recti muscles. No other morbid appearances observed.

*Remarks.* The facts brought to light on dissection were certainly unforeseen by most present. It was generally imagined that rupture of the bladder had taken place, an opinion apparently confirmed by the catheter's bringing away bloody urine. It is now pretty evident, that the instrument did not enter the bladder itself, but passed through the laceration in the urethra into the cellular membrane. This, too, accounts for the nature of the fluid drawn off. At first it was almost pure blood, because blood only was extravasated; but latterly the urine predominated in consequence of its escaping through the rent in the urethra, and mixing with the blood effused. The excessive pain over the pubes was manifestly owing to the infiltration of the urine into the cellular tissue, and its taking on the sloughy state. The patient died, however, more from the prostration consequent on so serious an accident, than from inflammation, for the skin was cold, and the pulse small from the commencement.

---

6. ANEURISM AT THE BEND OF THE ARM.

In our last number but one, we reviewed a case of this kind reported in the Lancet from the Westminster Hospital. In that journal Mr. White was censured for having put a ligature on the *radial* artery for a *varicose* aneurism. We observed that, from the perusal of the case, we were confident that the reporter was ignorant of the meaning of the terms he used, and that the *brachial* artery was tied for *diffused* aneurism. In a subsequent number of the Lancet our statement was asserted to be utterly false! In our last we again took up the subject, and proved that the falsehood lay—not with ourselves. In the Medical and Physical Journal for June, the case is related at large, by Mr. White himself. Into the details, of course, we shall not go, so much having been said on the subject already, but we may remark that they bear out our statements most completely; they prove that the aneurism *was* "diffused," and "clearly show that the brachial artery, (not the radial or ulnar, as has been erroneously stated) had been wounded." This is enough.

---

7. INCONTINENENCE OF URINE.

Two cases were lately mentioned at the Royal Academy of Medicine, by M. Canin, where dry-cupping the perineum, and a blister to the sacrum cured incontinence of urine in boys, one of 14 years of age, the other of 16 years. The former had been affected for two years with this complaint. He required eighteen applications of the cupping-glasses, in the course of a month. The cure was thus effected. In the second case, it required twenty applications, and a blister in addition. Various other means had been tried, in both cases, without effect.

---

8. ERGOT OF RYE.

M. Chevreul, a physician at Angers, has addressed a Memoir to the Royal Academy of Medicine, detailing sixteen cases in which the secale cornutum had been administered, in doses varying from 24 to 30 grains, as a mean of hastening delivery. Generally in the space of ten or fifteen minutes, pains of a peculiar character came on, and the labour was soon over. No sinister accident happened, in any of these cases, either to mother or infant.

---

9. UNSUSPECTED ABSCESSES IN THE LIVER.

[La Pitié.]

*Case* 1. Ant. Cyprien, a voiturier, aged 40 years, of strong constitution, after being a month in one of the surgical wards of La Pitié, for the treatment of some hæmorrhoidal tumours, was transferred to the medical wards, on the 26th May, for a quotidian intermittent fever, of irregular accession and duration. The patient presented an incoherence of ideas, and a stupor which could not be easily accounted for. The cheeks were flushed, and the action of the heart was tumultuous; but he complained of no pain in any part of the abdomen, which was supple, and void of tenderness. Constipation was obstinate, and cephalalgia intense. This state continued, without change, for nine days, when he died.

On dissection, a large abscess (four inches in diameter) was found in the centre of the great lobe of the liver, full of well formed pus. All the other viscera of the body were perfectly sound.

*Case* 2. A few months previously a case somewhat similar occurred in the same hospital, where an enormous abscess was found in the liver of a man who presented no symptom, while living, of such a malady being in progress. In this case the digestive organs were greatly disturbed in their functions, sympathetically, as were the functions of the brain in the preceding instance.

---

10. CAS RARES—REMARKABLE CASES.

In a former number of this Journal we gave an abstract of the celebrated article in the *Dictionnaire des Sciences Medicales*, entitled "CAS RARES," in which there were numerous cases, not only curious but useful. Haller was the first to keep a separate account of "rare cases," in his note book, and several physicians have since imitated the practice, especially Boehner. Dr. Cassan has lately published, in the ARCHIVES GÉNÉRALES, an article under this head, of which we shall take some notice in this place.

1. *Albinism.* A female, 33 years of age, was cited before the Chamber of Peers, to give evidence respecting the celebrated criminal Louvel. The effect of this citation was such that, in one night, her hair became completely blanched, while an extensive dartrous eruption came out on the head, forehead, and front of the chest. The eruption disappeared in time, but the whiteness of the hair remained permanent. There are several other instances of this kind on record, and among others, we believe, that of the late Queen of France.

2. *Rapid Formation of a Goitre.* Miss Louiset, after a violent corporeal exertion, in raising a weight, experienced, in 24 hours, a great enlargement of the left lobe of the thyroid gland—which enlargement has continued ever since.

3. *Expulsion of a Tænia.* A man was tormented with tape-worm for ten years, and tried all means of expelling the enemy, but without the least effect. He then despaired of success, and determined to live on friendly terms with this obstinate tenant of his nether region. One day, having eaten most voraciously of a soup made with fat pork, he was seized with a violent indigestion, during which the tænia was discharged entire.

4. *Permanent Hoarseness.* A young girl, who had begun to menstruate, was seized with a severe catarrh, for which she was bled from the arm, a few hours after the menstrual period had commenced. The catamenia were immediately suppressed, and she quickly lost her voice. A hoarseness succeeded, and has never since been removed. This is the third instance of a similar kind, from bleeding at an improper period, which our author has known.

5. *Great Dilatation of the Cardiac Veins.* A young lady, who was a teacher at a boarding-house, having experienced some severe mental affliction, became affected with orthopnœa, attacks of suffocation, irregularity of pulse, great emaciation, œdema of the legs and feet, dry cough, slight discharges of blood from the lungs, lancinating pains under the left false ribs. She removed to a Maison de Santé, and there died.

*Dissection.* The lungs were found healthy. The heart was of extraordinary dimensions, and its cavities filled with black blood. The two coronary veins were so enlarged, that the fore-finger could be readily introduced into them for some inches. The left cavities of the heart were greatly dilated, but their parietes were not extenuated. The right chambers were equally dilated, but here the walls were remarkably thin.

6. *Inequality of Pulse in the two Radials.* This phenomenon is by no means uncommon; but we cannot always recognise the cause after death. In the following case, however, the cause appears to have been revealed by dissection.

A female died of pneumonia, the pulse having been intermittent, irregular, small, and scarcely sensible in the right radial artery; while in the left arm the pulsations were strong, full, and regular. On dissection, the right lung was found completely hepatised, and pressing on the subclavian artery of that side. The left lung was free from disease.

11. ACUPUNCTURE.

Mr. Earle lately employed this mysterious remedy in a case of obstinate sciatica, which had resisted every other method of treatment. Two needles were introduced, to the depth of an inch, near the sacrum, and kept there a quarter of an hour. The sciatica almost immediately ceased, and the patient passed a quiet night, for the first time during some months. The old enemy returned, though not in force, a few days afterwards, and was finally routed by a couple of needles.

12. NITRATE OF POTASH IN MENORRHAGIA.

Dr. G. B. Carrese has lately published four cases of obstinate menorrhagia, cured by half-drachm doses thrice a-day. In all these cases, there had been mental troubles, attended with occasional suppressions and irregularities of the menstrual secretion. At length, the discharge was habitually so great as to injure the health, and then it was that Dr. C. administered the nitre in the above-mentioned doses, well diluted in barley-water, or other ptisans. In all the patients, the medicine produced a sense of coldness in the stomach, constriction, some nausea, and giddiness in the head. To these effects were added, an indescribable sense of tumult or revolution in the abdomen. The menorrhage in all the four cases, was soon cured. *Journ. Complem. Nov.* 1826.

13. AMAUROSIS.

The following history is given in Hufeland's Journal, for September last, by Dr. Hausbrond, of Bramesberg. Five children of a Jew merchant had had scarlatina, and were all convalescent, except a fine intelligent boy of 13 years of age, who did not recover so well as the others, though, in his case, the scarlatina had been milder. In the night of the 14th December, the boy became extremely restless, without apparent cause, then got delirious, and had several attacks of epileptiform convulsions. Dr. H. being called in, considered the child to be in a most dangerous situation, and that he would quickly expire. Three hours afterwards, however, a messenger informed the Doctor that the boy was rather better. The breathing had now become freer, the pulse perceptible, the animal heat was returning, and the features resuming something of their natural expression. When re-action had taken place, leeches to the temples, blisters to the nape of the neck, &c. were employed, while some calomel was exhibited internally. In the morning, the patient seemed deprived of his mental faculties, and of the sense of hearing; but these were partly restored after the leeches, blisters, &c. had acted. His speech, however, was greatly embarrassed. It was now discovered, also, that the boy was totally blind. The pupils were moderately dilated, and evinced no sensibility to light. Cold was now no longer applied to the head—and the antimoniated ointment was rubbed over the scalp. In the course of a little time, the antimonial pustules not only covered the head, like a crust of tinea favosa, but appeared on various other parts of the body, to which the antimony never had access. The sight returned at the end of five days, and the boy rapidly convalesced. When he was supposed to be entirely out of danger, at the end of five weeks, he became affected with a violent fever, which the Doctor considered as the secondary fever of the artificial pustules on the head and other parts of the body. The fact was, that the poor boy was brought into a dreadful state of irritation by the antimonial eruption, which harrassed him day and night. The patient, however, recovered from this fever, and his sight was perfectly restored.

---

14. INCISIONS IN ERYSIPELAS PHLEGMONODES.

We see, by a report from Bartholomew's Hospital, that Mr. Lawrence supports the method of treatment by incisions. A man had received a blow by a fall on the elbow. Erysipelatous inflammation followed, involving not only the skin, but the cellular membrane of the arm. Much constitutional fever and irritation were set up, and Mr. Lawrence resorted to incisions, carried the whole length of the inflamed parts, one being ten, and the other twelve inches in length. The exposed cellular membrane was found inflamed and thickened, and some dusky effusion, with a mixture of pus, was evolved from one of the incisions. The wounds bled freely, and all redness of the arm soon disappeared. Although some sloughing of the cellular membrane and integuments ensued, with considerable constitutional disturbance, yet the patient did well, and quickly recovered.—*Lancet, No.* 186.

---

15. NOXIOUS EXHALATIONS.

The experience of hospital surgeons must often have shewn the danger of bringing abraded surfaces within the range of exhalations from foul ulcers. The following is an instance.

A female was admitted into Bartholomew's, with an inflamed ulcer of the leg. Rest, leeches, and proper diet, soon brought the sore into a healing condition. When the cicatrization was considerably advanced, two other patients, one with mortification of the leg, and the other with a phagedenic ulcer of the foot, were placed in the adjoining beds. Quickly the first patient's sore took on an unhealthy aspect, and rapidly spread into an extensive sloughing ulcer. She was now removed into an airy ward, and nothing particular was done, in order to ascertain the influence of removal from the sphere of vitiated effluvia. The pain was immediately relieved, and the surrounding inflammation quickly subsided. But, as the surface did not seem inclined to clear, cinnabar fumigation was employed for a week, when the sloughy character disappeared, and the ulcer healed rapidly.

We hope the chloruret of lime may prove serviceable upon such occasions, by correcting the effluvia from foul sores, and preventing their action on others exposed to their influence.

---

16. NÆVI MATERNI.

Among the novelties of medical practice, we may mention a curious remedy for nævi materni, first employed, we understand, by Mr. Hodgson, of Birmingham, and now in course of trial by some surgeons of London. It is VACCINATION

of the nævus, in several points of its surface, by the specific inflammation of which, it is said, the nævus is arrested in its progress, or caused to slough. We recommend our surgical brethren to try this easy and simple remedy.*

We apprehend, however, that no plan will be equal to that of the ligature. If the tumour be too large for a single ligature to surround, a needle should be passed under the centre of the nævus with a double ligature, and then the two halves surrounded in the usual way. We believe that Mr. Lawrence ties the ligature very tight, and cuts it away at the end of 48 hours, to lessen irritation. A great number of nævi, of various sizes, have been removed in this manner by metropolitan surgeons, of late, without a single bad consequence.

* It has recently succeeded under Mr. Earle, at Bartholomew's.

---

